# Auto-Antibodies to β-F1-ATPase and Vimentin in Malignant Mesothelioma

**DOI:** 10.1371/journal.pone.0026515

**Published:** 2011-10-17

**Authors:** Jenette Creaney, Ian M. Dick, Deborah Yeoman, Sarah Wong, Bruce W. S. Robinson

**Affiliations:** 1 National Centre for Asbestos Related Diseases, University of Western Australia, School of Medicine and Pharmacology, Nedlands, Western Australia, Australia; 2 The Australian Mesothelioma Tissue Bank, Sir Charles Gairdner Hospital, Nedlands, Western Australia, Australia; 3 Department of Respiratory Medicine, Sir Charles Gairdner Hospital, Nedlands, Western Australia, Australia; King's College London, United Kingdom

## Abstract

Patients with Malignant Mesothelioma (MM) develop unidentified auto-antibodies to MM tumour antigens. This study was conducted to identify the targets of MM patient auto-antibodies in order to try to understand more of the anti-tumour response and to determine if these antibodies might be helpful for diagnosis or prognostication. Using MM patient sera in a Western immunoblott screening strategy, no common immunoreactive proteins were identified. The sera from one long-term survivor recognised a protein band of 50–60 kDa present in cell lysates from four of five MM cell lines tested. The immunoreactive proteins in this band were identified by 2D electrophoretic separation of a MM cell line protein lysate, followed by analysis of excised immunoreactive proteins on a MALDI TOF mass spectrometer and peptide mass fingerprinting. The immunoreactive proteins identified were vimentin (accession gi55977767) and the ATP synthase (F1-ATPase) beta chain (accession gi114549 and gi47606749). ELISA assays were developed for antibodies to these proteins. Neither vimentin (median and 95% CI 0.346; 0.32–0.468 for MM patients, 0.327; 0.308–0.428 for controls) nor ß-F1-ATPase (0.257; 0.221–0.453 for MM patients, 0.263; 0.22–0.35 for controls) showed significant differences in autoantibody levels between a group of MM patients and controls. Using a dichotomized antibody level (high, low) for these targets we demonstrated that vimentin antibody levels were not associated with survival. In contrast, high ß-F1-ATPase antibody levels were significantly associated with increased median survival (18 months) compared to low ß F1 ATPase antibody levels (9 months; p = 0.049). Immunohistochemical analysis on a MM tissue microarray showed cytoplasmic staining in 28 of 33 samples for vimentin and strong cytoplasmic staining in14 and weak in 16 samples for ß-F1-ATPase. Therefore antibodies to neither vimentin nor ß-F1-ATPase are useful for differential diagnosis of MM, however high antibody levels to ß-F1-ATPase may be associated with increased survival and this warrants further investigation.

## Introduction

New clinical biomarkers are needed for malignant mesothelioma (MM), an aggressive, asbestos-induced incurable tumour. The disease is difficult to diagnose and even with the best available treatments, patients have a median survival of less than a year after diagnosis and only 1% of patients survive five years [1], [2].

There has been a resurgence of interest in biomarkers for MM. Most interest has focussed on protein antigens, with mesothelin being the most promising. Mesothelinhas a sensitivity of 84% at a specificity of 95% in advanced MM [3], although sensitivity falls to 50% at the time of diagnosis [4] and to 15% in pre-diagnosis serum [5]. Other markers including megakaryocyte potentiating factor (MPF), osteopontin, CA125, CA15-3 and hyaluronic acid have been evaluated alone and in combination with mesothelin [4], [6], [7], [8], [9], [10] and no, or only minimal, improvements of diagnostic sensitivity over mesothelin have been observed. Therefore new and/or novel candidate biomarkers for MM diagnosis need to be identified and evaluated.

Rather than focussing on new antigens, another approach to discovering biomarkers has been to identify anti-tumour auto-antibodies. During tumourigenisis considerable molecular changes result in increased and/or aberrant production, altered post-translational modification and altered cellular distribution of proteins. This complex suite of abnormal protein expression, structure and distribution can potentially result in the generation of a complex auto-antibody profile in individual patients [11]. Auto-antibodies against autologous tumourassociatedantigens have been detected in many types of cancer including lung cancer [12], [13].

Previously using the serological identification by recombinant expression cloning (SEREX) approach [14] we identified tumour associated antigens recognised by MM patient sera, the majority of specificities were uniquely associated with individual patients though some common reactivities were observed including against topoisomerase IIβ, U2AF(65) [15] and also ß-F1-ATPase (unpublished data).

Using an one dimensional Western immunoblotting screening strategy we have previously demonstrated that some MM patients exhibit high titre antibodies to MM proteins expressed on cultured MM cell lines [16]. However in the previous study there was no commonly recognised antigenic pattern for MM patients - indeed at the level of sensitivity of western blotting, patients primarily appeared to have “private”, rather than “public” specificities [16]. In this study we used a different approach, identifying antigenic proteins intensely recognised by Western immunoblotting of a patient with a good prognosis for MM and then determining, using the more sensitive and specific ELISA methodology, whether in a larger group of MM patients the presence of these antibodies might be useful in diagnosis or indicative of prognosis.

## Results

### Auto-antibody profile in MM patients

**Figure 1:**
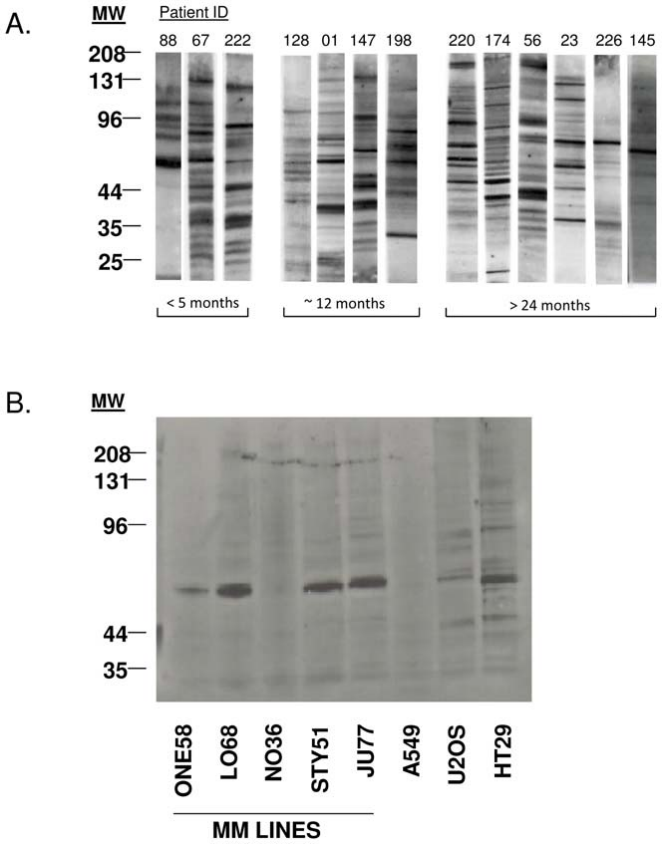
Western Immunoblots of MM patient serum with MM tumour cell line lysates. (A) Representative serum samples from individual MM patients. Patients segregated into short, medium and long term survivors. Patient identification number indicated above each lane. (B) Western immunoblot of cell lysates from five different MM cell lines and the A549 lung adenocarcinoma, U2OS osteosarcoma and HT29 colon carcinoma cell lines incubated with serum from a MM Patient 145. Serum was diluted 1∶100. Molecular weights (MW) depicted to the left of figures.

The auto-antibody profile of serum samples collected from approximately 150 MM patients within two months of diagnosis was analysed by one dimensional Western immunoblotting against total protein lysates from MM cell lines. Individual MM patients recognised specific protein regions on the membrane at varying intensities and in the majority of cases patients exhibited multiple reactivities(Figure 1A). As previously reported [16] there was no common antibody reactivity for MM patients. No obvious correlation was observed between the number or intensity of reactivities and the overall patient group survival.

One individual (Patient ID #145), a 73 year old woman with pleural epithelial MM demonstrated strong IgG reactivity to a protein(s) of between 50 and 60 kDa in her sera taken two months post-diagnosis. This individual was alive 45 months after MM diagnosis. The immunoreactive protein(s) was present in the total cell lysates of four of five of the MM cell lines examined and also in the lysates of the bone osteosarcoma (U2OS) and the colon carcinoma (HT29) cell lines. No immunoreactivity was detected against proteins present in the MM line (NO36) or the lung adenocarcinoma line (A549) (Figure 1B). The immunoreactivity was evident when sera were titrated out to 1∶1600 (data not shown). Fractionation of the MM cell line, JU77, demonstrated that the immunodominant protein(s) was present within the Tris-soluble fraction (data not shown).

### Identification of immunoreactive proteins

**Figure 2:**
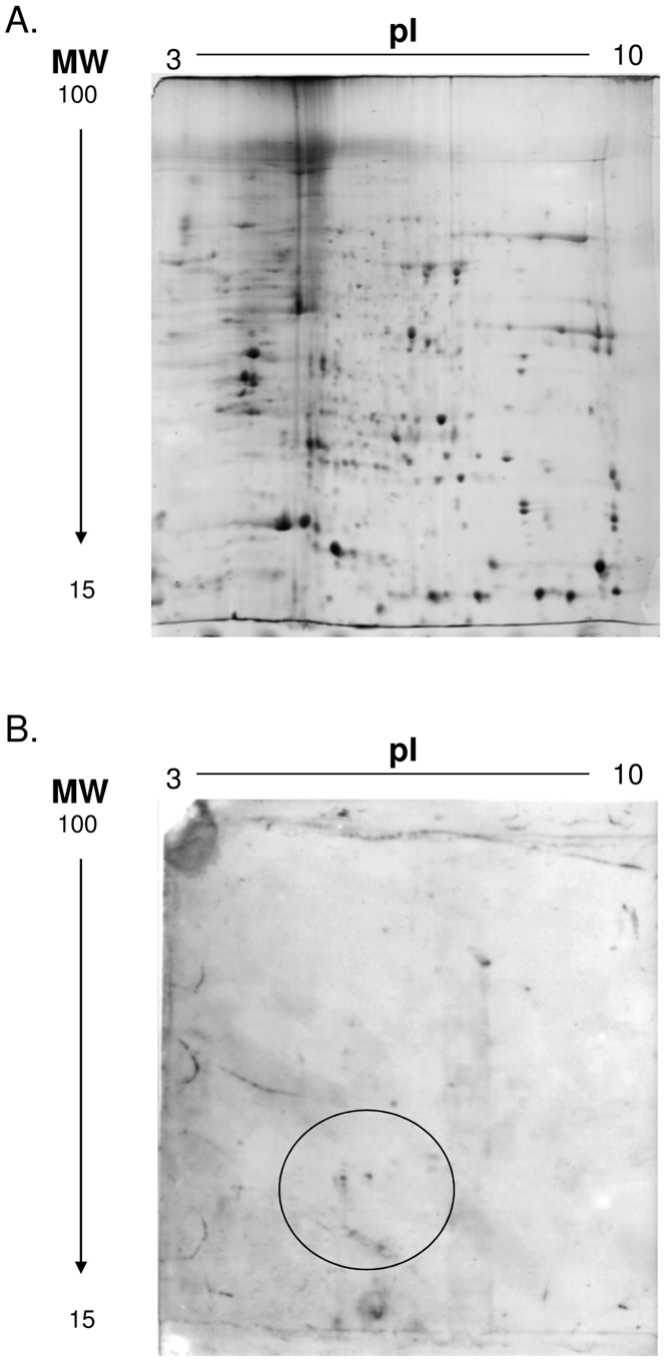
Two Dimensional Polyacrylamide Gel Electrophoresis. The Tris-soluble fraction of the JU77 cell lysate was separated by 2D electrophoresis and proteins stained with Coomassie Blue. Proteins from a parallel gel were transferred to PDVF and incubated with serum from MM Patient 145 diluted 1∶100. Two densely stained IgG reactive spots were defined by the approximate pI range 5 to 7 and molecular weight range 50 to 60 kDa, indicated by the circle. Molecular weights (MW) depicted to the left of figures; isoelectric point (pI) to the top of figures.

**Figure 3:**
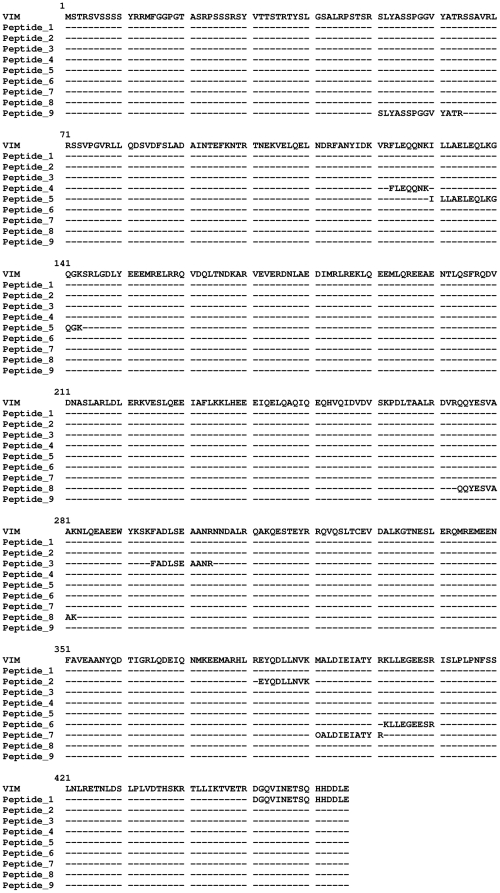
Sequence alignment of mass peaks matching to Vimentin. Clustal alignment of the Vimentin sequence with trypsin digestion peptides identified by MS with a matching sequence of confidence 85% or greater.

**Figure 4:**
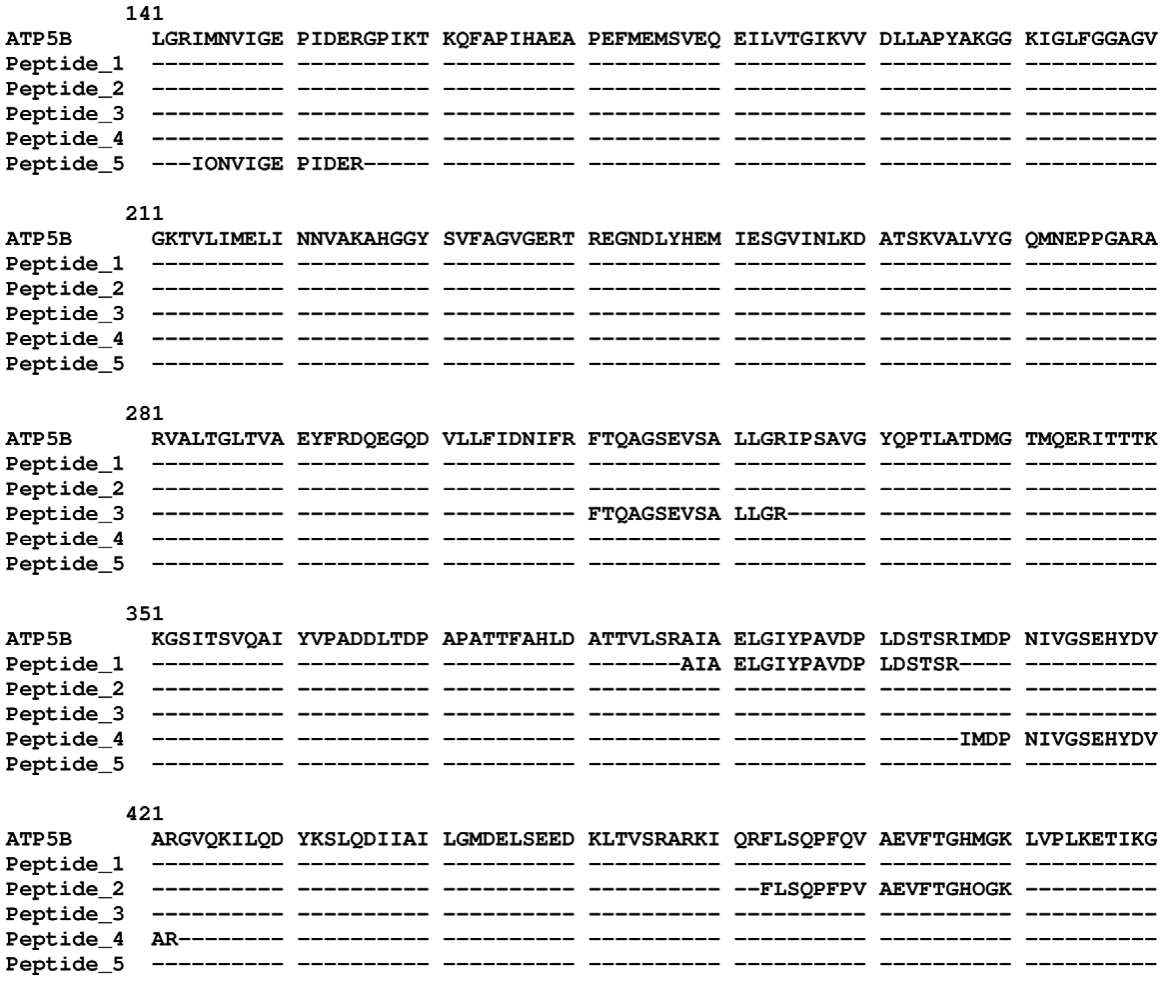
Sequence alignment of mass peaks matching to ATP5B. Clustal alignment of the ATP5B sequence with trypsin digestion peptides identified by MS with a matching sequence of confidence 85% or greater.

To identify the immunodominant protein(s) the Tris-soluble fraction of JU77 cells wasseparated by two dimensional-electrophoresis and electrophoreticly transferred to PVDF membranes, before immunostaining. Two densely stained regions were observed, defined by the approximate pI range 5 to 7 and molecular weight range 50 to 60 kDa(Figure 2). The regions corresponding to the immunodominant spots were identified from a parallel G250 Coomassie stained gel and four samples excised from the gel. Following peptide sequencing by mass spectrometry (MS) two of the samples gave statistically significant matches, vimentin (VIM) (Accession no. gi55977767) with nine matches (Figure 3) and ATP synthase beta chain, mitochondrial precursor (ATP5B) (Accession no. gi 114549 and gi47606749) with nineteen and twelve matches (Figure 4).

In this study validation experiments were attempted using immunoblotting in denaturing conditions however Patient #145's sera did not recognise recombinant human or bovine vimentin in Western immunoblotting, and immunoreactivity to MM cell extract was not inhibited by competitive binding experiments (data not shown). Similarly, although ß-F1-ATPase was recognised as an antigen by two other patients in a SEREX screening strategy (unpublished observations), the clone/recombinant protein which contained an open reading frame corresponding to the C-terminal portion of the protein (nt 595–1736) was not recognised by the Patient #145's sera by Western immunoblotting. These results are likely to be due to the effect of the denaturing conditions on the auto-antibody epitopes [17].

### Tissue localisation of vimentin

**Figure 5:**
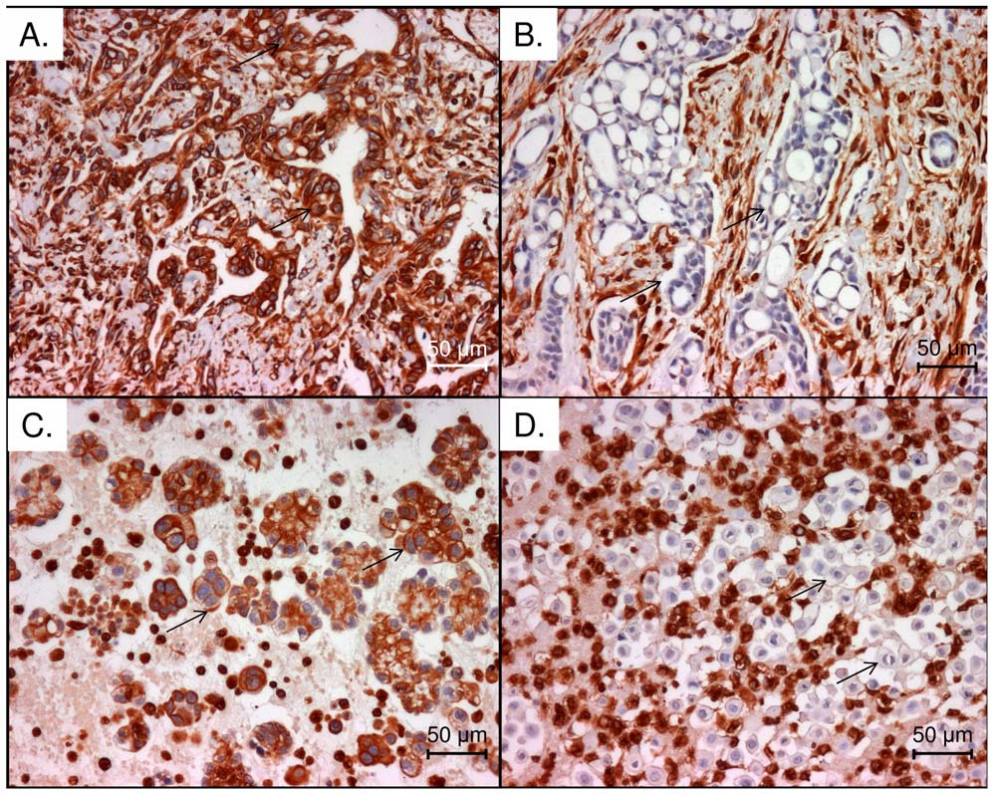
Immunohistochemicallocalisation of vimentin. Immunohistochemical staining of representative MM samples using anti-vimentin antibody diluted 1∶500 (A) Positive stained MM tumour sample, arrows indicate representative groups of tumour cells; over 90% of the tumour cells have been stained (B) Non stained MM tumour sample with positively stained stroma. Arrows indicate representative groups of non-staining tumour cells; less than 10% of tumour cells have been stained. (C) Positively stained MM cells present in a pleural effusion sample. Arrows indicate representative positive stained MM cells; over 90% of the tumour cells have been stained. (D) Non stained MM tumour cells in a MM patient pleural effusion. Arrows indicate representative non-staining tumour cells; less than 10% of tumour cells have been stained. Photomicrographs were taken at ×200 magnification.

To confirm that MM cells express vimentinimmunolocalisation experiments were conducted on independent MM patient samples. Vimentin was detected in the cytoplasm of tumour cells, non-malignant stroma and blood vessel endothelial cells. Of the 34 MM tissues examined, in duplicate, 24 stained intensely positive for vimentin, 4 cases were weakly or equivocally stained and 6 were negative. Similarly of the 24 cases of MM effusions, tumour cells were positively stained in 19 cases, weakly stained in 1 case and 4 were negative (Figure 5).

### Frequency of anti-vimentin antibodies in MM patients

**Figure 6:**
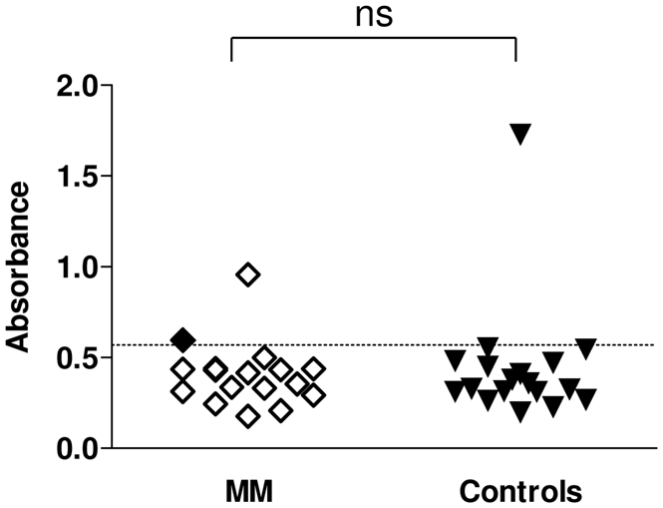
Vimentin ELISA. Levels of antibodies to vimentin were determined at least in duplicate and individual patient (open diamonds) and control (closed triangles) values are plotted on the graph. The defined cut-off value of two standard deviations above the mean of the control group (excluding the outlier) is depicted by the hashed horizontal line. The index MM patient sample (Patient 145) result is indicated by the closed black diamond symbol. There was no significant difference (ns) between the groups.

An ELISA system was developed using human recombinant vimentin as an antigen and antibody levels were determined in a small group of MM patients and healthy controls (Clinical characteristics presented in Table 1). The majority of control subjects with no asbestos exposure had anti-vimentin antibodies present with an absorbance below 0.69. One control individual had high titre reactivity. Excluding the elevated control subject, the median (95% CI) serum anti-vimentin levels of the MM patients (0.346; 0.32–0.468) was not significantly different to that of controls (0.327; 0.308–0.428). Excluding the elevated control subject, two of the MM patients had antibody levels above the threshold defined by the control group (ie mean+2 standard deviations of the mean) including the index patient in which the original reactivity to vimentin was demonstrated (Figure 6).

**Table 1:** Clinical characteristics and antibody levels for study subjects.

Clinical Data	Malignant Mesothelioma		Controls
	Short term survivors^1^	Long term survivors^2^	
Number	11	11	20
Mean Age (range)	67 (52–82)	63 (42–82)	52 (43–66)
Sex (M∶F)	11∶0	10∶1	16∶4
Asbestos Exposure			
Yes	7	8	1
No	-	-	8
Unknown	4	3	11
Histology			
Epitheliod	5	8	-
Biphasic	1	-	-
Not specified	5	3	-
Median Survival (range)	8 (3–11)	28 (14–58)	-
Anti-vimentin^3^ (OD)	0.33 (0.1–0.81)	0.42 (0.31–0.46)	0.34 (0.27–0.61)
Anti-β-F1-ATPase^3^ (OD)	0.18 (0.14–0.43)	0.27 (0.19–0.59)	0.27 (0.23–0.38)

1 – Short term survival group; survival <12 months.

2 – Long term survival group; survival >12 months.

3 - median ± 95% confidence intervals.

### Tissue localisation of ß-F1-ATPase

**Figure 7:**
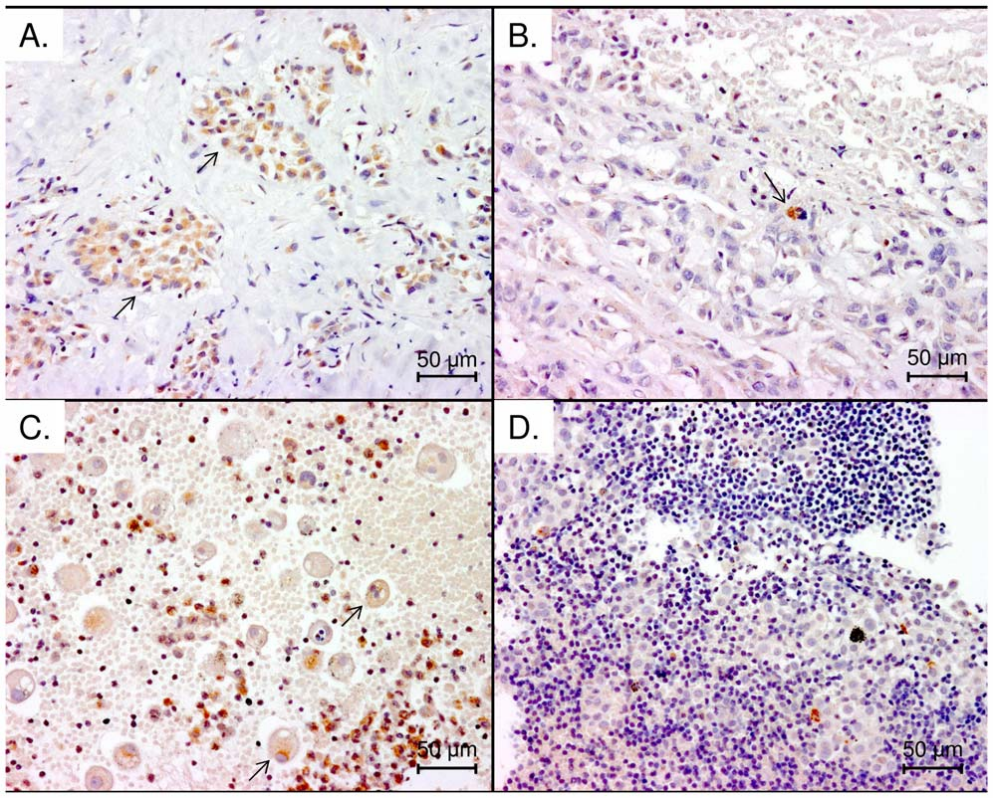
Immunohistochemical tissue localisation of β-F1-ATPpase. Immunohistochemical staining of representative MM samples using anti-β-F1-ATPpase antibody diluted 1∶100 (A) Positive stained MM tumour sample, arrows indicate representative groups of tumour cells; over 90% of the tumour cells have been stained. (B) Non stained MM tumour sample with positively stained macrophage (indicated by arrow); less than 10% of tumour cells have been stained. (C) Positively stained MM cells present in a pleural effusion sample. Arrows indicate representative positive stained MM cells; over 90% of the tumour cells have been stained. (D) Non stained MM tumour cells in a MM patient pleural effusion; less than 10% of tumour cells have been stained. Photomicrographs were taken at a ×200 magnification.

ß-F1-ATPase stained the cytoplasm of MM cells in tissue sections, in some cells staining was accentuated in the perinuclear region. Of the 34 MM tissue samples examined, 14 stained positive for ß-F1-ATPase, 16 cases were weakly or equivocally stained and 4 were negative. Of the 21 cases of effusion from MM patients, tumour cells were weakly positively stained in 14 cases, and negative in 7 cases (Figure 7). There was strong staining for ß-F1-ATPase in macrophages present in the tumour infiltrate and in effusions, but it was unclear if this staining was of the macrophage mitochondria or of phagocytosed material within the cytoplasm of the macrophage.

### Frequency of anti-ß-F1-ATPase antibodies in MM patients

**Figure 8:**
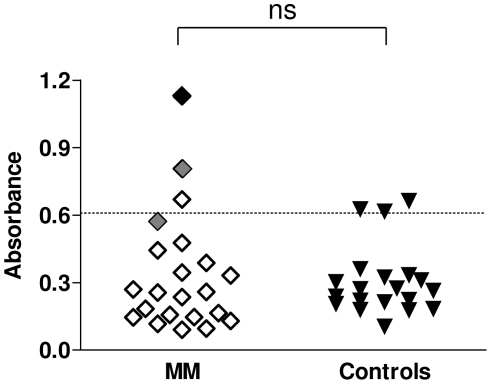
ß-F1-ATPase ELISA. Levels of antibodies to ß-F1-ATPase were determined at least in duplicate and individual patient (open diamonds) and control (closed triangles) values are plotted on the graph. The defined cut-off value of two standard deviations above the mean of the control group is depicted by the hashed horizontal line. The index MM patient sample (Patient 145) result is indicated by the closed black diamond symbol. The MM patients indicated by grey symbols were previously identified as being immunopositive to ß-F1-ATPase by SEREX. There was no significant difference (ns) between the groups.

There was no significant difference in the level of antibodies to ß-F1-ATPase in the group of MM patients (median = 0.257; 95% CI 0.221–0.453) relative to control subjects (0.263; 0.22–0.35). However four patients had antibody levels greater than 2 standard deviations above the mean of the control population, including the patient in whom the anti-ß-F1-ATPase antibodies were originally identified in, and one of the SEREX positive patients (Figure 8).

### Prognostic significance of anti-vimentin and ß-F1-ATPase antibodies

**Figure 9:**
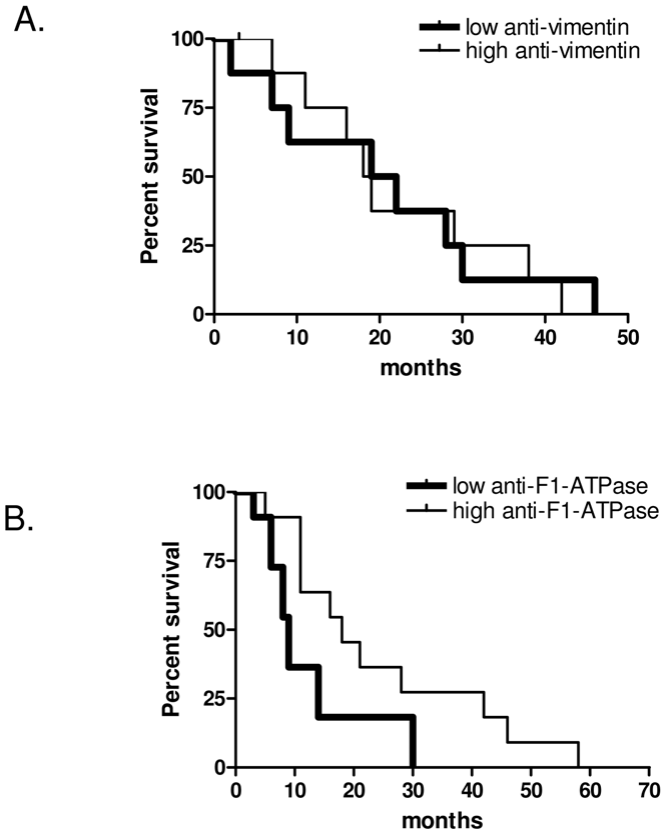
Survival of MM patients dichotomized on antibody reactivity. Kaplan–Meier survival curves of overall survival (months) from diagnosis for patients with (A) anti-vimentin and (B) anti-β-F1-ATPase antibody levels dichotomized below (thick line) and above the median (thin line).

Antibodies to vimentin and to ß-F1-ATPase were identified from the sera of an individual who survived 43 months from diagnosis. No survival advantage was demonstrated when patients were dichotomised into having high or low concentrations of anti-vimentin antibodies in the sera (Figure 9A). The group of patients with high levels of anti-ß-F1-ATPases in the serum had a median survival of 18 months, which was significantly longer than the group with low antibody levels (median survival 9 months; p = 0.049) (Figure 9B).

## Discussion

Using an immuno-proteomic approach two candidate autoantibody biomarkers for MM were identified; vimentin and ß-F1-ATPase. Vimentin has previously been evaluated as an immunohistochemical marker in MM and to our knowledge ß-F1-ATPase is a novel marker in this disease.

Vimentin is an intermediate filament characteristic of mesenchymal tissue, and has been extensively examined for a potential role as an immunohistochemical marker for MM tissue diagnosis. Reports of vimentin expression in MM range from 16% to 100% (reviewed in [18]). In our small series 74% of MM cells in tissue and effusions express the protein. As a significant percentage of lung adenocarcinomas also express the protein it is generally concluded to be of little value for differential diagnosis for these diseases [18]. However, as the majority of MM tumours express the protein this may be an explanation for the inducement of anti-vimentin antibodies observed in MM patients in this study.

Antibodies to vimentin have been observed in several different autoimmune conditions including rheumatoid arthritis and systemic lupus erythematosus [19] and also following solid-organ transplantation [20]. In this present study levels of antibodies to this protein were not significantly higher in the group of MM patients examined relative to apparently healthy persons, although one individual, a 55 year-old man with a history of moderate autoimmune disease had an elevated antibody level. The autoimmune status of the MM patients, which may have contributed to anti-vimentin antibody production, was not known, and of course the identification of antibodies to vimentin may have been an epiphenomenon.

The second protein identified was the β-subunit of F1-ATPase. The archetypical ATP synthase is located in the mitochondrial membrane and is comprised of several subunits. The catalytic β subunit is the rate-limiting component for ATP production. In cancer, a common feature is a shift from oxidative phosphorylation to glycolytic metabolism, known as the Warburg effect [21], and this is associated with a down regulation of mitochondrial ATP synthase [22], which has been observed in a variety of different tumours including colorectal cancer [23], breast cancer [24] and lung adenocarcinoma [25]. The mechanism by which ATP synthase is down regulated has not been fully elucidated; though a specific inhibitory protein has been identified [26]. Low expressionof ß-F1-ATPase has been associated with shorter survival times in colorectal cancer patients [27].

In this study ß-F1-ATPase expression was demonstrated in the cytoplasm of less than half of the MM tumours examined. Whether a specific tumour expresses the protein may depend on the degree of oxygenation of the tumour or of the sampled area of the tumour. In addition to being located in the mitochondria, ß-F1-ATPase has been shown to be present on the cell membrane of endothelial cells and tumours of various cell types [28]. Under the conditions of the current study no surface expression of ß-F1-ATPase was observed, however it is possible that cell surface ß-F1-ATPase was masked by tumour-expressed MHC-I antigens [29]. It is not known if anti- β-F1-ATPase antibodies observed in the present study were generated because the protein was over expressed or expressed in a different context.

Recently antibodies to ß-F1-ATPase have been demonstrated in about a third of patients with Alzheimer's disease [30]. None of the healthy donors or patients with Parkinson's disease or atherosclerosis had antibodies though a small number of individuals with systemic lupus erythematosus and multiple sclerosis were positive in this assay. In *in vitro* assays these auto-antibodies induced cell death by apoptosis. It would be of interest to determine if patients with other conditions and malignancies also develop antibodies to this protein.

ß-F1-ATPase was recognised by the sera of a MM patient with a relatively good survival outcome (45 months), in our cohort of MM patients less than 3% survive for longer than this time (data not shown). We found that whilst anti-ß-F1-ATPase antibody levels were not significantly elevated in the group of MM patients relative to controls; the group of MM patients with relatively high levels of antibodies to ß-F1-ATPase had a survival advantage over those with low antibody levels. Whilst the presence of antibodies to ß-F1-ATPase may be a secondary marker of prognois, it is interesting to speculate on a more direct role for this protein and antibodies directed at it. One possibility is that the presence of ß-F1-ATPase antibodies was indicative of a higher expression of non-membranous ß-F1-ATPase and that a higher cytoplasmic expression of this protein is associated with an improved survival outcome, as is the case for colorectal cancer [27]. Alternatively, these antibodies may be directed toward cell surface ß-F1-ATPase. This would be of interest because a number of *in vitro* studies have shown that targeting cell surface expressed F1 ATPase by antibodies [28], [31] or by an ATPase inhibitor reduces cell proliferation by inducing apoptosis and arresting cell cycle at the G0/G1 check point [32] which may translate to a survival advantage in patients. Although not clearly associated with antibody levels, ß-F1-ATPase bound to apolipoprotein-A-1 on the tumour cell surface has been demonstrated to be a target for the cytolytic action of gamma-delta T cells [33].

In summary, this study used a two dimensional immuno-proteomic approach to identify antigens recognised by sera from a MM patient with a relatively good survival outcome. Vimentin and ß-F1-ATPase were identified as autoantigens but only anti-ß-F1-ATPase correlated with survival. These data add to our knowledge of the role of ß-F1-ATPase in cancer and warrant its further investigation as a prognostic marker and potential therapeutic target in MM.

## Materials and Methods

### Patient samples

Samples were collected from patients presenting to the respiratory clinics of either Sir Charles Gairdner Hospital or the Hollywood Specialist Centre in Perth, Western Australia, and from healthy laboratory volunteers. This study was approved by the human research ethics committees of Sir Charles Gairdner and Hollywood Hospitals and all subjects provided written informed consent. The samples form part of the Australian Mesothelioma Tissue Bank, a member bank of the Australasian Biospecimen Network which is supported in part by the Australian National Health and Medical Research Council. The final diagnosis in all patients was confirmed by pathologists experienced in the diagnosis of MM.

Blood samples were collected by routine venepuncture and allowed to clot for at least 2 h at room temperature, or at 4°C overnight before processing. Samples were centrifuged at 1,200 rpm for 10 minutes, then the supernatant was removed, aliquoted and stored at −80°C until use.

### Cell Lines

The human malignant mesothelioma cell lines, ONE58, LO68, NO36, STY51 and JU77 were all derived from the pleural fluid or tumours of MM patients essentially as described by Manning et al. [34]. These cell lines have been deposited in CellBank Australia (Westmead, NSW) and found to be free of contamination and confirmed to be unique through short tandem repeat profiling.

The human lung adenocarcinoma, A549, colon cancer line HT29 and bone osteosarcoma U2OSwere purchased from the American Type Culture Collection (Manassas, VA). Cells were cultured in RPMI-1640 (Life Technologies, Melbourne, Australia) supplemented with 20 mM HEPES, 5×10^−5^ M 2-mercaptoethanol, 10% foetal calf serum (Life Technologies) and incubated at 37°C in a 5% CO_2_ humidified atmosphere. All cells were harvested when they were about 70–85% confluent.

### SDS-Polyacrylamide Gel Electrophoresis and Western Immunoblotting

Total protein lysates from cell lines were prepared as previously described [16]. Fractionation of cells was performed as follows, approximately 2×10^8^ JU77 cells were trypsinised from tissue culture flasks, washed and resuspended in ice-cold 40 mM Tris (pH 7.4) supplemented with protease inhibitors (Boehringer Mannheim, Germany) and sonicated using a Branford Sonifier (450) probe sonicator. Cellular DNA was sheared by passing the sample through a 21 gauge needle, followed by further sonication. Samples were centrifuged at 20,000 *g* at 4°C for 15 min and the Tris-soluble fraction stored at −20°C. The resultant pellet was resuspended in 10 mM HEPES for 15 minutes on ice, Nonidet P-40 was added to a final concentration of 1.25% and the sample vortexed for 30 sec, before being subjected to a further centrifugation step. The resultant HEPES soluble fraction was stored at −20°C and the pellet resuspended in 40 mM Tris and lysed by the addition of 2×SDS loading buffer.

Protein was separated by molecular weight on 10% Tris-HCl gels (BioRad Laboratories, CA). Protein was transferred to nitrocellulose membrane and Western immunoblotting was performed as described [35]. Briefly membranes were blocked in 10% skim milk powder and incubated with patient sera diluted 1∶100 overnight at 4°C. Bound antibody was detected following incubation with goat anti-human IgG conjugated to horse radish peroxidase (Sigma, NJ) and ECL chemiluminescence reagent (GE Healthcare, NJ).

### Two Dimensional Polyacrylamide Gel Electrophoresis

Proteins within the tris soluble fraction of the equivalent of 10^7^ JU77 cells were separated by 2D polyacrylamide electrophoresis essentially as described [36] Briefly, the tris-soluble fraction was methanol-precipitated and resuspended in a solubilisation solution consisting of 5 M urea, 2 M thiourea, 2 mM n-tributyl phosphine, 0.5% pH 3–10 Pharmalyte carrier ampholytes (Pharmacia, Uppsala, Sweden), 2% 3-((3-cholamidopropyl), dimethylammonio)1-propanesulfonate (CHAPS), 2% caprylylsulfobetaine, 0.001% Orange G dye and endonuclease. Protein was separated in the first dimension using Pharmacia Immobiline immobilized pH-gradient (IPG) 18-cm linear gradient strips at various pI ranges. Samples were separated in the second dimension on denaturing 10% polyacrylamide slab gels (16×18 cm). For each sample two gels were run in parallel, proteins in the first gel were visualised using 1% Commassie Brilliant Blue G250 (BioRad) and proteins in the second gel were transferred to polyvinylenedifluoride membrane (BioRad) for subsequent immunoblotting as described above. Two dimensional polyacrylamide gel electrophoresis was performed by Proteomics International (Nedlands, Western Australia).

### Peptide-Massfingerprinting

Protein spots were excised from stained gels by hand and processed for peptide mass fingerprinting as described [36]. Briefly, following tryptic digestion peptides were analysed in a PerSeptiveBiosystems Voyager DE-STR (Framingham, MA, USA) matrix-assisted laser desorption/ionization time of-flight (MALDI-TOF) mass spectrometer. The peptide masses were then used to search the SWISS-PROT and TrEMBL databases using the program PeptIdent (http://expasy.proteome.org.au/tools/peptident.html).

### ELISA

ELISA were optimised in preliminary experiments and performed essentially as described [35]. Wells of a 96-well plate (MAXISORB, Nunc) were coated with either recombinant human vimentin (Research Diagnostics, NJ) at 5 µg/ml or yeast ß-F1-ATPase (a kind gift from Andrew Rodgers, University of Western Australia) at 1 µg/ml in 100 mMNaHCO_3_, pH 9.6 overnight at 4°C. Wells were washed three times with PBST, blocked for 1 h in 1% bovine serum albumin (Sigma) and serum samples were assayed in duplicated at three dilutions 1∶50, 1∶100 and 1∶200. Following overnight incubation and washing, bound antibody was detected with alkaline phosphatase conjugated anti-human IgG (Promega). The wells were incubated with pNPP substrate and the optical densities (OD) of the wells measured. A positive antibody response was defined as an OD reading of two standard deviations or greater above the mean. The ELISAs were highly reproducible and variance between duplicates did not exceed 8%.

### Immunohistochemistry

Immunohistochemistry was performed using standard techniques on atissue microarray of 33 MM tumour tissue samples, 26 MM pleural effusions and a range of malignant and normal tissues including tonsil, kidney, liver, appendix and colon as described previously [4]. Primary antibodies, anti-vimentin (Ab-1, clone V-9, Oncogene, San Diego, CA) and anti-ATP synthase subunit β (anti-F_1_F_0_-ATPase subunit β) (clone 3D5, Molecular Probes, Eugene, OR) were used at a dilution of 1∶500 and 1∶100 respectively. A positive result was defined as the presence of stain in more than 33% of tumour cells as determined by morphology and staining on parallel slides with calretinin and EMA. Staining intensity was graded semi-quantitatively as negative, weak (1+), moderate (2+) or strong (3+).

### Statistical Analysis

For comparison between two groups a Mann Whitney test was applied. Patient survival was analysed using the Kaplan Meier product limit procedure and patients were stratified into groups based on whether they expressed high or low antibody levels, defined as being above or below the median value for antibodies for a particular protein. A p value of less than 0.05 was considered significant. Statistical analysis was carried out using the SPSS version 18 statistical package (IBM Somers NY).
